# Impact of insecticide-treated nets on wild pyrethroid resistant *Anopheles epiroticus *population from southern Vietnam tested in experimental huts

**DOI:** 10.1186/1475-2875-8-248

**Published:** 2009-10-29

**Authors:** Wim Van Bortel, Vu Duc Chinh, Dirk Berkvens, Niko Speybroeck, Ho Dinh Trung, Marc Coosemans

**Affiliations:** 1 Institute of Tropical Medicine, Dept Parasitology, Nationalestraat 155, B-2000 Antwerpen, Belgium; 2 National Institute of Malariology, Parasitology and Entomology, Dept Entomology, Luong The Vinh street, B.C. 10.200 Tu Liem, Hanoi, Vietnam; 3 Institute of Tropical Medicine, Dept Animal Health, Nationalestraat 155, B-2000 Antwerpen, Belgium; 4 Department of Biomedical Sciences, Faculty of Pharmaceutical, Veterinary and Biomedical Sciences, University of Antwerp, Universiteitsplein 1, B-2610 Antwerpen (Wilrijk), Belgium

## Abstract

**Background:**

In this study, the efficacy of insecticide-treated nets was evaluated in terms of deterrence, blood-feeding inhibition, induced exophily and mortality on a wild resistant population of *Anopheles epiroticus *in southern Vietnam, in order to gain insight into the operational consequences of the insecticide resistance observed in this malaria vector in the Mekong delta.

**Method:**

An experimental station, based on the model of West Africa and adapted to the behaviour of the target species, was built in southern Vietnam. The study design was adapted from the WHO phase 2 guidelines. The study arms included a conventionally treated polyester net (CTN) with deltamethrin washed just before exhaustion, the WHO recommended long-lasting insecticidal net (LLIN) PermaNet 2.0^® ^unwashed and 20 times washed and PermaNet 3.0^®^, designed for the control of pyrethroid resistant vectors, unwashed and 20 times washed.

**Results:**

The nets still provided personal protection against the resistant *An. epiroticus *population. The personal protection ranged from 67% for deltamethrin CTN to 85% for unwashed PermaNet 3.0. Insecticide resistance in the *An. epiroticus *mosquitoes did not seem to alter the deterrent effect of pyrethroids. A significant higher mortality was still observed among the treatment arms despite the fact that the *An. epiroticus *population is resistant against the tested insecticides.

**Conclusion:**

This study shows that CTN and LLINs still protect individuals against a pyrethroid resistant malaria vector from the Mekong region, where insecticide resistance is caused by a metabolic mechanism. In the light of a possible elimination of malaria from the Mekong region these insights in operational consequences of the insecticide resistance on control tools is of upmost importance.

## Background

Insecticide resistance has been demonstrated in many African and Asian malaria vectors [1,2]. The impact of the observed resistance on the applied control tools will depend on the mechanism(s) conferring resistance, the biology of the vector and the control method applied. Evidence exists that resistance reduces the efficacy of indoor residual spraying (IRS). In South Africa, for example, increased levels of mixed function oxidases in *Anopheles funestus *was associated with malaria control failure [3]. On the island Bioko, Equatorial Guinea, IRS with pyrethroids failed to reduce the pyrethroid resistant *Anopheles gambiae sensu stricto *population, whereas IRS using carbamates was effective in the following spraying rounds [4]. The situation is more complex with insecticide-treated nets, that are acting at the same time as a physical and chemical barrier. A high *kdr *frequency in *An. gambiae s.s*. populations of Ivory Coast had no effect on the effectiveness of pyrethroid-treated nets [5-7]. However, in the neighbouring country Benin, insecticide-treated nets failed to control pyrethroid resistant *An. gambiae *[8].

Most research has been focussing on the situation in Africa, whereas the situation in Asia is not studied. In the Mekong region a clear picture of insecticide resistance status of malaria vectors was achieved after a three years intense insecticide resistance survey in the framework of the MALVECASIA network [9]. In Laos, Cambodia and Thailand, insecticide resistance in the malaria vectors *Anopheles dirus s.s., An. epiroticus *and *Anopheles minimus senso lato *was almost absent, whereas in Vietnam resistance was found in the malaria vectors *An. minimus s.l*. in some northern localities and in *An. epiroticus *in the Mekong delta. The latter species was found to be resistant to all pyrethroid insecticides tested in the Mekong delta. It was susceptible to DDT, except in the suburbs of Ho Chi Minh City where it showed DDT tolerance. Studies on the mechanism of insecticide resistance revealed that knockdown target site resistance does not occur in the major vectors, *An. minimus s.l*, *An. dirus s.l*. and *An. epiroticus*. Biochemical assays indicate increased levels of esterase activity in pyrethroid resistant populations of *An. epiroticus*. A high esterase activity was found alone or in combination with an elevated level of P450 monooxygenases in *An. minimus s.s*. populations [10].

Insecticide resistance in Vietnam was only observed in low or transmission free areas and the MALVECASIA network concluded that there is no need changing the malaria vector control strategy currently implemented in Vietnam. However, the current malaria situation in this area is a consequence of the implementation of the comprehensive national malaria control programme including pyrethroid treated bed net distribution [11-13]. Furthermore, access to effective preventive measures is essential to further contain malaria in endemic foci and vector control can play a role in restraining the spread of drug resistance of the *Plasmodium *parasite [14]. Consequently, because of the observed insecticide resistance in southern Vietnam there is an urgent need to assess operational consequences. For this purpose, experimental houses were constructed in southern Vietnam to evaluate the efficacy of existing vector control tools in terms of deterrence, blood-feeding inhibition, induced exophily and mortality on wild resistant population of *An. epiroticus*.

## Methods

### Study site

The study was carried out in Van Duc A village, Bac Lieu province (Mekong Delta), southern Vietnam (9.18353 N, 105.30339 E). In previous studies *An. epiroticus, Anopheles sinensis, Anopheles subpictus, Anopheles nimpe, Anopheles campestris *and *Anopheles vagus *were collected in the study site [15]. The main species of interest is *An. epiroticus*, which is also the most abundant. This population of *An. epiroticus *is resistant against deltamethrin, alpha-cypermethrin, etofenprox and cyfluthrin but susceptible to DDT [9]. Additional WHO tube bioassays [16] were done using propoxur 0.1% and malathion 5% at discriminative dosage to obtain a full picture of the insecticide resistance status of the species. In 2008 bioassays were done with deltamethrin 0.05% to assess differences in resistance status between 2005 and 2008.

### Hut construction

**Figure 1:**
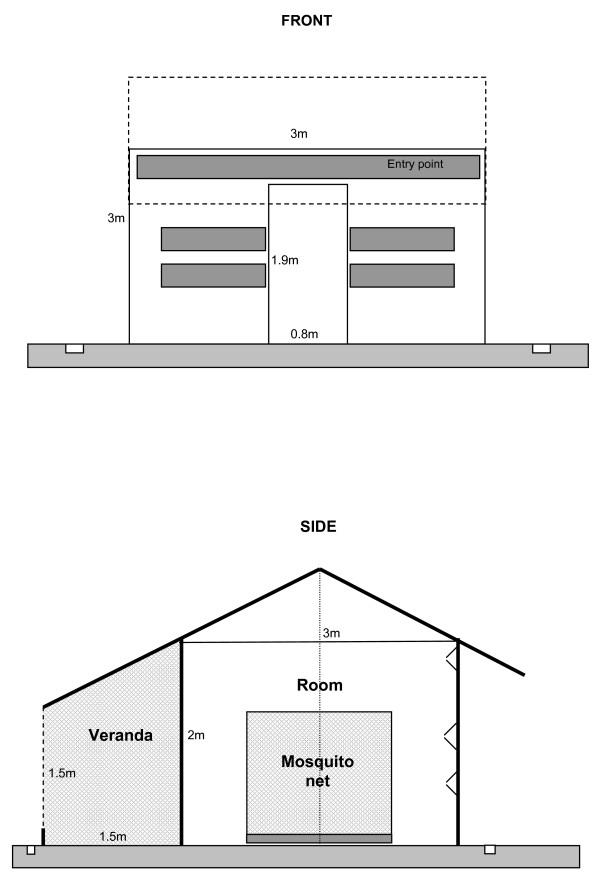
**Schematic presentation of the experimental huts built in the Mekong delta, southern Vietnam**.

Six experimental huts adapted from the huts used in West Africa were built using local construction materials (Figure 1) [17]. The huts were built on a concrete floor and had a wooden structure. The outside walls were covered with nipa-palm leaves. The lower parts of the inside walls were made of leaves and the upper part of plastic hessian sheeting. The roof was covered with palm tree leaves and inside with plastic sheeting. Each hut was surrounded by a water filled gutter to prevent the entry of scavengers such as ants. The entry side of the huts faced a large brackish water swamp. Entry slits were available in the front panel of the hut: two entry slits (0.75 m long) at each side of the door and one large slit above the door over the entire width of the front panel (3 m). These slits were designed to prevent mosquitoes from escaping once they entered the hut. The slits were covered by a plastic curtain from 5 a.m. till 6 p.m. Each hut was provided with a full screened veranda trap for collecting exophilic mosquitoes. The escape rate from the huts was evaluated by releasing 100 mosquitoes in each hut at 9 p.m. and re-capturing the mosquitoes in the morning from 6 till 8 a.m. The huts were adapted till at least 75% of mosquitoes were re-captured.

### Treatment arms

Unwashed and washed Permanet 2.0 and 3.0 were compared to a negative control (Table 1). Permanet 2.0 is a polyester LLIN coated with deltamethrin. PermaNet 3.0 is a complex long-lasting insecticidal net designed for the control of insecticide resistant mosquito populations. It comprises deltamethrin-coated-polyester side panels and a deltamethrin plus piperonyl butoxide (PBO) incorporated-polyethylene roof. The multifilament polyester (75 denier) yarns of the side panels have a deltamethrin load of 85 mg AI/m^2^. The fabric of the lower part (70 cm) is denser compared to the higher part resulting in deltamethrin concentrations of 115 mg AI/m^2^. The roof is made of 100 denier polyethylene monofilament containing 4 g AI/kg deltamethrin and 25 g/kg (± 25%) PBO by incorporation. The safety of PermaNet 3.0 has been reviewed by WHO, following the WHO generic risk assessment model for insecticide treatment of mosquito nets and their subsequent use and concluded that use and washing of these treated nets do not pose an undue hazard to the user [18].

Six holes each 4 cm × 4 cm were made in each mosquito net, two in each long side and one at each end, to simulate the conditions of a torn net and to put the emphasis on testing whether the insecticidal treatment, rather than the net, effectively prevents biting of the sleepers.

### Study design

The design was adapted from the WHO guidelines [19]. A base line study was conducted in December 2006 to evaluate the attractiveness of the six huts. The trial lasted for six weeks from 23 September till 28 October 2008 and treatment arms rotated weekly among the six experimental huts. Treatments were assigned randomly among huts in such a way that each treatment was tested once, during one week, in each hut according to a Latin Square Design. Six nets were used per treatment arm and each net was tested during one night of the week. Each morning the nets were removed and stored in their corresponding plastic bag. At the end of the week huts were cleaned and ventilated for one day before moving to another treatment. In each hut, a team of two adult volunteers slept under one net from 7 p.m. to 5 a.m. The six teams of volunteers rotated daily according to the Latin square design described above. Before the volunteers left the net at 5 a.m. they collected the dead and live mosquitoes inside the net. At 6 a.m., live and dead mosquitoes were collected from the hut (room) and from the veranda. Mosquitoes were identified using a standardized key for anophelines of Southeast Asia [20], counted and scored by hut and collection place as dead or alive, blood-fed, unfed or gravid. Live females were put in cups supplied with 10% sugar solution for 24 hours, after which any delayed mortality was recorded.

Ethical consideration: The protocol was approved by the ethical committees of the University Hospital of Antwerp (EC Nr 6/45/221) and of NIMPE Hanoi Vietnam. Volunteers gave written informed consent.

### Bioassay

Cone bioassays, following the WHO procedures [19], were carried out on one net per arm before washing the nets, just before the trial in case the nets were washed and after the trial. Bioassays were done at NIMPE-Hanoi using 2-5 days old unfed females of a full susceptible *An. dirus s.s*. colony strain kept at NIMPE-Hanoi for ten years which allowed to evaluate the bio-availability of the insecticides on the net. For each tested net 10 pieces (2 × 4 side panels, 2 × 1 roof) of net were tested resulting in 50 mosquitoes per net. Mosquitoes were exposed during three minutes. Knockdown was recorded 60 minutes after exposure and mortality 24 h post exposure and compared to the WHO criteria for LLIN i.e. > 80% mortality and/or > 95% knockdown [21]. For PermaNet 3.0 washed 20 times, 30 sections were tested (10 on the lower side panels below 70 cm, 10 on the higher side panels, 10 on the roof).

### Chemical analysis

Net pieces were analysed before washing, after washing before the trial and after the trial. The side panels and roof were tested separately. The chromatographic determination of deltamethrin, deltamethrin R-isomer and piperonyl butoxide was based on the CIPAC method 32+33+345/TK/(M)/3. The chemical analyses were performed by a WHO reference centre (Pesticides Research Department of the Walloon Agricultural Centre, Gembloux, Belgium).

### Statistical analysis

The outcomes measured in experimental huts were: (1) the entry rate or total number of mosquitoes found in the huts. This is used to measure the deterrent effect which is the reduction in hut entry relative to the control huts, (2) the exit rate measured as the proportion of mosquitoes found in the veranda, (3) blood feeding or proportion of blood fed female mosquitoes, (4) the mortality rate calculated as the proportion of dead mosquitoes, immediate and delayed observations combined, found in the huts. The percentage personal protection was calculated as (BF_C_-BF_T_)/(BF_C_) * 100, where BF_C _is the total number of blood fed females in the control hut and BF_T _the total number of blood-fed female mosquitoes in the treated hut. The overall killing effect of a treatment was calculated as (D_T_-D_C_)/(T_C_) * 100, where D_T _is the total number of dead mosquitoes in the treated hut, D_C _the total number of dead mosquitoes in the control hut and T_C _is the total number of mosquitoes collected in the control hut [19]. Statistical analysis of numbers (entry rates) was done by means of a negative binomial regression. The entry, blood feeding and mortality rates were compared between control arm and intervention arms by logistic regression (Stata 9).

## Results

### Baseline survey

The baseline survey lasted for six days in December 2006 during which 1,104 *An. epiroticus*, 24 *An. nimpe*, two *An. sinensis *and one *An. campestris *were collected. No difference in attractiveness was observed between the huts (Negative Binomial Regression, p > 0.05).

### Trial

**Figure 2:**
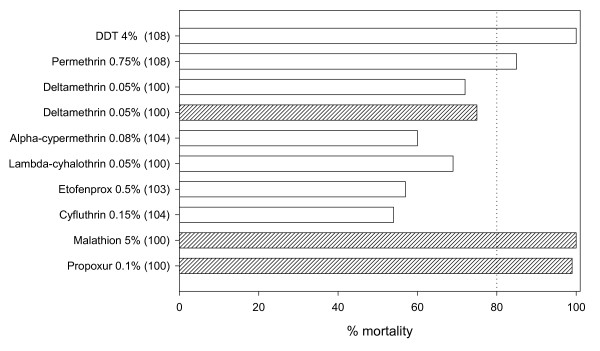
**Overview of the resistance status of *Anopheles epiroticus *from Bac Lieu, southern Vietnam**. The open bars are results from March 2005, bioassays done in the framework of the MALVECASIA project [9]. The hatched bars are results obtained during this study. Number between brackets corresponds to the number of tested mosquitoes. Dotted line (80%) is the WHO resistance threshold.

Mosquitoes were collected during 216 hut-nights (6 weeks × 6 days × 6 huts). from September till October 2008. In total 19,784 *An. epiroticus*, 400 *An. nimpe*, seven *An. sinensis *and 7,403 Culicinae were caught. Only *An. epiroticus *was further considered in the analyses of which the resistance status is given in figure 2.

Before any washing, mortality in cone bioassay was 100% for all treatments and knockdown was higher than 95%. Both PermaNet 2.0 and 3.0 showed mortality equal or higher than 98% after 20 washes and a knockdown of at least 86%. The deltamethrin CTN washed just before exhaustion (five washes) induced a mortality and a knockdown of respectively 90 and 94%. After the trial unwashed PermaNet 2.0 and 3.0 and the 20 times washed PermaNet 3.0 complied with the WHOPES criteria for LLINs, i.e. knockdown ≥ 95% and/or mortality ≥ 80%, whereas Permanet 2.0 was border line with a mortality of 78% and a knockdown of 88%. The deltamethrin CTN washed 5 times induced a mortality and a knockdown of only 24 and 38% respectively.

The active ingredient content for unwashed PermaNet 3.0 (deltamethrin and PBO) and PermaNet 2.0 (deltamethrin) complied with the target doses (± 25%), except for one unwashed net of PermaNet 3.0 for which the average deltamethrin content on the side panels (2.05 g AI/kg) was just below the lower limit (2.1 g AI/kg) of the target dose. For this same unwashed PermaNet 3.0, the relative standard deviation for the side panels was 12% (Table 1). The overall active ingredient retention for PermaNet 3.0 after 20 washes was 55% and 100% on the side panels and roof respectively and 72% for PBO on the roof. The overall deltamethrin retention for PermaNet 2.0 after 20 washes was 50%. The deltamethrin CTN had an initial concentration of 22.2 mg AI/m^2 ^which is in line with the target dose of 25 mg AI/m^2^. After five washes this decreased to 5.4 mg AI/m^2^.

**Table 1:** Overview of the treatment arms of the experimental hut trial in the Mekong delta, southern Vietnam.

			Actual Concentration^b ^mg AI/m^2 ^(%RSD)		Actual Concentration as % of the target concentration	
				
Treatment arm	Type of net^A^	Target Concentration of unwashed nets mg AI/m^2^	unwashed	20× washed (*5× washed)	unwashed	20× washed (*5× washed)
1	unwashed PermaNet 2.0	55	63.9 (2%)	NA	116%	NA
2	unwashed PermaNet 3.0	85 (upper part of the side panel)	81.6 (2%)	NA	96%	NA
3	20× washed PermaNet 2.0	55	66.0 (4%)	31.5 (3%)	120%	57%
4	20× washed PermaNet 3.0	85 (upper part of the side panel)	61.5 (12%)	33.6 (15%)	72%	40%
5	CTN, washed just before exhaustion	25	22.2 (13%)	5.4 (12%)*	89%	22%*
Control	Untreated polyester net	--	--	--		

^A ^CTN = Conventionally Treated Net, treated with Deltamethrin K-Othrin SC10, Bayer Crop Science. Surface of the nets: 14.2 m^2^

^b ^Actual content measured at the start of the trial on pieces from the side panel. Measured by the WHO reference centre 'Pesticides Research Department of the Walloon Agricultural Centre, Gembloux, Belgium'. %RSD, percentage relative standard deviation. NA, not applicable.

The estimated deterrent effect for *An. epiroticus *varied between 20 and 35% for washed or unwashed PermaNet 2.0 or 3.0, and was only 7% with the CTN washed just before exhaustion (Table 2). In huts with untreated nets, 43% of the mosquitoes were collected in the veranda, and exophily ranged from 35% to 54% in the treatment arms. Blood feeding inhibition varied between 64% and 81% in the treated arms. PermaNet 3.0 washed 20 times did not induce a significantly higher blood feeding inhibition than Permanet 2.0 washed 20 times (Table 2). The overall mortality was relatively high in the untreated control arm (34%), which was mainly due to high mortality of the unfed females. The overall mortality with the PermaNet arms was between 96 and 82% compared to 70% for the CTN. The overall mortality among blood fed in the untreated control arm was low (10%) and relatively high in all treatment arms (79-94%). There were no significant differences between the different PermaNet arms but mortality among blood fed mosquitoes was significantly lower with the CTN. The overall insecticidal effect varied between 30 and 43%, the latter observed with unwashed PermaNet 3.0 (Table 2).

**Table 2:** Results for *Anopheles epiroticus *of the experimental hut trial in the Mekong delta, southern Vietnam

**Outcomes measures**^A^	Control	PermaNet 2.0 unwashed	PermaNet 3.0 unwashed	PermaNet 2.0 20× washed	PermaNet 3.0 20× washed	CTN Washed just before exhaustion (5 × washed)
ENTRY RATE						
Total females caught	4114	2851	3289	3038	2671	3821
Females caught per night	114.3^a^	79.2^a, b^	91.4^a, b^	84.4^a, b^	74.2^b^	106.1^a, b^
Deterrence % (T_C _- T_T_)/(T_C_)*100	--	30.7	20.1	26.2	35.1	7.1
EXIT RATE						
Total Females in veranda	1757	1105	1185	1419	921	2043
Exophily %	42.7^a^	38.8^b^	36.0^c^	46.7^d^	34.5^c^	53.5^e^
95% CI	41.2 - 44.2	37.0 - 40.6	34.4 - 37.7	44.9 - 48.5	32.7 - 36.3	51.9 - 55.1
BLOOD FEEDING RATE						
Total females blood fed	999	189	150	236	178	334
blood fed (%)	24.3^a^	6.6^b^	4.6^c^	7.8^b, d^	6.7^b^	8.7^d^
95% CI	23.0 - 25.6	5.7 - 7.6	3.9 - 5.3	6.8 - 8.8	5.7 - 7.7	7.9 - 9.7
Blood fed inhibition % (%BF_C_-%BF_T_)/(%BF_C_)*100	--	72.7	81.2	68.0	72.6	64.0
personal protection % (BF_C_-BF_T_)/(BF_C_)*100	--	81.1	85.0	76.4	82.2	66.6
MORTALITY RATE						
Total females dead	1394	2617	3145	2487	2437	2645
Overall mortality (%)	34.1^a^	92.2^b^	96.4^c^	82.3^d^	91.6^b^	69.7^e^
95% CI	32.7 - 35.6	91.1 - 93.2	95.7 - 97.0	80.9 - 83.6	90.5 - 92.6	68.2 - 71.1
Overall insecticidal effect (D_T_-D_C_)/(T_C_)*100	--	29.7	42.6	26.6	25.4	30.4
Overall mortality among blood fed (%)	10.1^a^	89.9^b^	94.7^b^	93.6^b^	92.0^b^	81.2^c^
95% CI	8.3 - 12.1	84.7 - 93.8	98.8 - 97.7	89.6 - 96.4	87.0 - 95.6	76.6 - 85.3
Overall corrected mortality among blood fed (%)	--	88.8	94.1	92.8	91.2	79.1

Number in the same row sharing a letter superscript do not differ significantly *(P *> 0.05). Significance level for difference *P *< 0.05 ^A^Tc: total number of mosquitoes collected in control arm; T_T_: total number of mosquitoes collected in treated arm; BF_C_: total number of blood fed females in the control arm; BF_T_: total number of blood-fed female mosquitoes in the treated arm; D_C_: total number of dead mosquitoes in the control arm; D_T _: total number of dead mosquitoes in the treated arm.

## Discussion

An experimental station was built in southern Vietnam to evaluate the operational consequences of the insecticide resistance found in the malaria vector *An. epiroticus*. The design of the experimental huts was based on the model of West Africa [17] and adapted to the behaviour of the target species. Therefore, the numbers of entry points were increased but still more than 75% of mosquitoes were re-captured after a release experiment to evaluate the escape rates from the huts. The six huts were built in front of breeding sites of *An. epiroticus *and were equally attractive for mosquitoes. The huts were constructed using local material that is commonly used for the construction of houses in the Mekong delta.

This study showed that unwashed and 20 times washed LLINs and five time washed CTN still provided personal protection against an *An. epiroticus *population found to be pyrethroid resistant as defined by the WHO tube bioassay. The deliberately holed nets still acted as a barrier to mosquitoes searching for blood meal and the insecticidal treatment, rather than the net, effectively prevented mosquitoes to bite sleepers. The personal protection ranged from 67% for deltamethrin CTN washed 5 times to 85% for unwashed PermaNet 3.0. This is only slightly lower than the personal protection observed in northern Benin with susceptible *An. gambiae *mosquitoes [8]. All PermaNet arms were performing almost equally or slightly better than conventionally treated nets washed until just before exhaustion and no substantial difference was observed for the different performance indicators between PermaNet 2.0 and 3.0. Insecticide resistance in the *An. epiroticus *mosquitoes did not seem to alter the well-known deterrent effect of pyrethroids, unfortunately baseline data on the deterrent effect of susceptible *An. epiroticus *is missing in the Mekong region.

*Anopheles epiroticus *showed high exophilic behaviour even in the control huts, corroborating previous observations on the behaviour of this *Anopeheles *species [15]. Many of these exophilic mosquitoes were found unfed and showed a high mortality in the untreated and treated arms making a correct interpretation of the overall insecticidal effect unreliable. Though, the mortality among fed mosquitoes in the control hut was only 10%. A significant higher mortality was still observed among the treatment arms despite the fact that the *An. epiroticus *population is resistant against the tested insecticides. Likewise in Ivory Cost high mortality was still observed in experimental huts with a resistant *An. gambiae *population [6,22] and the protective efficacy of nets remained high [7]. In this area of Africa, *kdr *is the main insecticide resistance mechanisms involved and it has been shown that *kdr *changes the irritability of the resistant mosquitoes resulting in a longer exposure to the insecticide [23]. *Kdr *is however absent in *An. epiroticus *of the Mekong region [10] and metabolic resistance is believed to induce a loss of efficacy [3]. The resistant status of *An. epiroticus *from Vietnam clearly contrasts with the one observed in Thailand and Cambodia where this species was found to be fully susceptible based on the same discriminative dosage [9]. Though, the observed pyrethroid resistant levels in Vietnam as measured by WHO bioassays (mortality between 50-80%) might not by high enough to induce an operational impact. Hence, it might be expected that PermaNet 3.0, designed for controlling insecticide resistant populations, will not have an additive impact in this context. However, the additional value of PermaNet 3.0 is questionable as different field studies did not show an increased killing effect on resistant *Culex *mosquitoes [18].

## Conclusion

Experimental houses were constructed in southern Vietnam to evaluate the efficacy of existing vector control tools in terms of deterrence, blood-feeding inhibition, induced exophily and mortality on wild resistant populations of *An. epiroticus*. This study showed that unwashed and washed treated bed nets still provided personal protection against pyrethroid resistant *An. epiroticus *mosquitoes. Malaria control in the Mekong Region is confronted with a different situation compared to Africa. Malaria is still present in endemic foci whereas large areas are malaria free. In this context elimination seems to be a realistic option [24]. Vector control will play an important role in containing malaria in these endemic foci and can play a role in restraining the spread of drug resistance by decreasing the circulation of the resistant parasites in the population. This can only be achieved when control tools are effective. Hence, there is an urgent need for more insight in the metabolic based resistance mechanisms, the operational consequences of the insecticide resistance on control tools as well as a better understanding of the dynamics and trends over time of loss of efficacy of LLINs in the presence of resistant vectors. Experimental hut trials are an essential step to test the operational implications of the insecticide resistance detected by WHO bioassays which can be studied by National Control Programmes.

## Competing interests

The authors declare that they have no competing interests.

## Authors' contributions

WVB, MC and HDT designed the experimental houses, initiated the study and revised and supervised the study critically at all stages. WVB carried out the data analysis and drafted the manuscript. VDC facilitated and carried out the field work and did the data entry. NS and DB assisted to the study design. All authors contributed to the manuscript and approved the final manuscript.
